# Influence of climate and geography on the occurrence of *Legionella* and amoebae in composting facilities

**DOI:** 10.1186/1756-0500-7-831

**Published:** 2014-11-24

**Authors:** Lisa Conza, Simona Casati Pagani, Valeria Gaia

**Affiliations:** Swiss National Reference Centre for Legionella, Bellinzona, Switzerland; Servizio di microbiologia EOLAB, Ente ospedaliero cantonale, Bellinzona, Switzerland

**Keywords:** *Legionella*, Legionnaires’ Disease, Free-living amoebae, Bioaerosol, Compost, Climate, Meteorological factors

## Abstract

**Background:**

The incidence of Legionnaires’ disease (LD) in southern Switzerland is three times higher than in northern Switzerland. Climatic and geographic factors may be potential causes for this difference.

We studied the prevalence of *Legionella* and free-living amoebae (FLA) in compost and bioaerosol in two Swiss regions to understand the role of climate and geography in the transmission of LD. We also tried to investigate whether or not compost storage duration would influence the composition of *Legionella* and FLA communities.

**Results:**

A larger proportion of compost heaps in facilities from southern Switzerland harbor more diverse *Legionella* compared to the north (*P* = 0.0146). FLA were isolated from composts in northern facilities at slightly higher rates (88.2% vs. 69.2%) and at lower rates from bioaerosols (6.3% vs. 13%) than in southern Switzerland. The diversity of FLA was higher in northern than in southern Switzerland (80% vs. 65%). A general decrease in the presence and variety of species was observed with decreasing compost storage time length. A discriminant model showed that values of vapour pressure, relative humidity and temperature distinguish the two regions, which were also characterised by different contamination rates by FLA and *Legionella*.

**Conclusions:**

The duration of outdoor storage may favour contamination of the compost by *Legionella*, and may increase the number and isolation of *Legionella* naturally occurring in compost. The climate in the south seems to favour higher *Legionella* contamination of compost heaps: this could explain the higher incidence of LD in southern Switzerland.

**Electronic supplementary material:**

The online version of this article (doi:10.1186/1756-0500-7-831) contains supplementary material, which is available to authorized users.

## Background

Legionnaires’ disease (LD) is caused by inhalation of bioaerosols arising mainly from water sources containing species of *Legionella*[1, 2], which often survive and grow within free-living amoebae [FLA; e.g. *Acanthamoeba* spp., *Vermamoeba* spp. (synonym of *Hartmannella*) and *Naegleria* spp. [3]] in water, soils and composts [4]. FLA are thought to play an important role in the survival of *Legionella* in compost heaps and as dispersal vehicles in bioaerosols [5, 6].

In-house water systems and cooling towers are considered the main, but not the only source of community-acquired LD [1, 7]. In Europe, infection sources are still unknown for 52.4% of the outbreaks [2]. Soils, potting soils and composts have been associated with LD cases caused by *L. longbeachae* and by *L. pneumophila*[8–10]*.* Potting soils and composts have also been described as reservoirs of *Legionella* also in Switzerland [11, 12].

In 2006-2010, the incidence of LD in Europe was about 1 case per 100,000 inhabitants per year with a mortality rate of 6.6% [2, 13]. In Switzerland, in the same period, the incidence was similar, being approximately 1-3 cases per 100,000 inhabitants per year with a mortality rate of 6.5% [14]; most of the cases were community acquired, sporadic and not equally distributed over the whole territory. The incidence in many Swiss Cantons corresponds to the Swiss average [14]; by contrast, in the Canton Ticino, southern Switzerland, the incidence is about 10 cases per 100,000 inhabitants per year [14]. This high incidence could be caused to some extent by a more accurate reporting system or by a higher proportion of patients with pneumonia tested for *Legionella*, but other as yet unknown factors may be involved.

Climate could play an important role in the transmission of LD from sources located within the community. Switzerland is divided by the Alps that act as a barrier and create two distinct climatic zones [15]. The North of the Alps has a prevalently humid, continental climate, characterised by cold winters with little precipitations and warm summers with frequent precipitations. The South, on the other hand, is characterised by mild, wet winters, warm to hot and dry summers, and heavy rain in autumn/winter [16, 17], as well as by a particular geomorphology (“Insubric climate”) [18]. The lowlands are located in valleys with lakes that favour dry winters with a relative humidity that at times may be as low as 20% [18]. The summer is normally dry, with heavy thunderstorms and short, heavy spells of rainfalls. Heavy and frequent rainfalls are common during the period from June to September (approx. 800–1,200 mm), with a mean annual precipitation of 1,600-2,100 mm, in contrast to the dry summers of the Mediterranean climate [18]. The marked differences in climate between the northern and southern part of Switzerland could be a reason for the different incidence of LD in both regions. On the other hand, it cannot be excluded that differences in *Legionella* and FLA populations in composts and bioaerosols from the two regions may also play a role in the LD transmission. This study has thus been designed to investigate whether composting facilities in southern and northern Switzerland host different populations of *Legionella* and FLA. We analysed composts and bioaerosols released from facilities located in the two regions for the presence of *Legionella* and FLA. We collected atmospheric data and measured the temperature of the composts in all facilities studied. We also carried out a detailed investigation of different composting facilities in the Canton Ticino to investigate whether or not the duration of compost storage might influence the composition of *Legionella* and FLA communities.

## Methods

### Sampling sites

Sampling sites are listed in Table 1. We studied eight open field composting facilities (CF), of which four (CF1-CF4), located in the Canton Ticino, were sampled between May and August 2009, whereas the other four (CF5-CF8), in the Swiss northern alpine regions (Cantons of Zurich, Aargau and Lucerne) were investigated between May and August 2010 (Table 1). Open-field CF are characterised by a structured management of the green waste that are regularly mixed until maturation [11], during which bioaerosols are produced [19]. We also investigated five communal outdoor green waste centres in Ticino. Three short-term storage (CS1-CS3) and two long-term storage centres (CL1-CL2) were sampled between May and September 2008. Both types of centres collect green waste and store the material in piles. In short-term storage centres the green waste is stored for a maximum of three months, whereas in the long-term centres it is kept between three months to two years before redistribution. All CF, short-term and long-term storage centres are distributed randomly over the territory and collect green waste from different areas of the region.

**Table 1:** **Sampling locations and number of samples (CS; short-term green wastes storage centres, CL; long-term green wastes storage centres, CF; composting facilities)**

Facility	Region ^***a***^	Canton	No. of heaps ^***b***^	Location description	No. of samples	
					Bioaerosol	Compost
CF1	S	Ticino	15-20	Open lowland area	4	2
CF2	S	Ticino	4-5	Closed area in a wood on a hilly terrain	3	1
CF3	S	Ticino	4-5	Open lowland area	4	2
CF4	S	Ticino	15-20	Closed area in a wood on a hilly terrain	4	2
CF5	N	Zurich	> 30	Open lowland area	4	4
CF6	N	Zurich	4-5	Open lowland area	4	4
CF7	N	Aargau	15-20	Closed area in a wood on a hilly terrain	4	4
CF8^*c*^	N	Lucerne	>30	Closed area in a wood on a hilly terrain	4	5
CL1	S	Ticino	2-3	Open lowland area	2	2
CL2	S	Ticino	2-3	Open lowland area	2	2
CS1	S	Ticino	1-2	Open lowland area	1	1
CS2	S	Ticino	2-3	Open lowland area	2	0
CS3	S	Ticino	1-2	Open lowland area	1	1

^*a*^S, southern alpine region; N, northern alpine region; ^*b*^No. of compost heaps per facility; ^*c*^Partially indoor facility.

### Sample collection

Bioaerosol samples were collected in different spots of all facilities, starting from the steaming compost piles up to a maximum distance of 5 m from the piles (green waste, ground material, middle stage and final compost). The air samples (1 m^3^) were collected in 10 ml Page’s saline solution (PAGE) with a portable cyclonic air sampler (Coriolis μ, Bertin technologies, Montigny, France), as described previously [20]. *Legionella* and FLA isolation was carried out within 24 h after collection.

### Compost processing

Compost samples (approx. 1 kg) were collected from the steaming piles at a depth of about 30 cm, stored in plastics bags and conserved at 4°C; within 24 hours from collection, a 5 g compost portion was suspended in 10 ml sterile PAGE and processed as described by Casati and co-workers [12].

### Identification of free-living amoebae

FLA were isolated from composts and bioaerosols as described by Conza et al. [21]. Non-nutrient agar plates were inoculated with 40 μl of the homogenised supernatant of the composts [11] or 50 μl of the concentrated bioaerosols and incubated at 28°C. When FLA growth was observed, the trophozoites were gently scraped from the surface of the plates and suspended in 3 ml PAGE. Genomic DNA from FLA was extracted using the DNeasy kit (Qiagen, Hombrechtikon, Switzerland) following the manufacturer’s instructions. FLA were identified by sequencing using the primers Ami6Fdeg and Ami9R [22]. The sequences obtained were compared with those available in GenBank using BLAST.

### Culture of *Legionella*from composts

Compost samples were analysed as described by Casati et al. [11]. Isolation of *Legionella* was carried out after acid treatment (0.2 M HCl–KCl acid buffer) on modified Wadowsky-Yee (MWY) (Oxoid, Pratteln, Switzerland) and glycine-vancomycin-polymyxin B-cycloheximide (GVPC) (bioMérieux, Geneva, Switzerland) selective media, to which propiconazole (Sigma-Aldrich, Basel, Switzerland) was added at a final concentration of 5 × 10^-4^ mg/ml to inhibit fungal growth. The limit of detection of culture for bioaerosol samples is high (10^6^ cells in 1 m^3^ air) [20], therefore bioaerosols were analysed by co-culture only.

### Co-culture of *Legionella*from compost and bioaerosol samples

All compost and bioaerosol samples were analysed by co-culture as previously described [20]. 900 μl of *Acanthamoeba polyphaga* were added to each well of a 24-well microplate (Techno Plastic Products AG, Trasadingen, Switzerland) and incubated for 1 h at 36°C to obtain an amoebal monolayer. 100 μl of 1 : 10^3^ diluted bioaerosols and compost suspensions (1 : 10^5^ dilution) were then inoculated into the wells; one well containing 900 μl of *A. polyphaga* suspension was added to 100 μl of sterile PAGE and used as a negative control. Aliquots of 20 μl were acid treated, spread on GVPC (bioMérieux) media, and incubated at 36°C for 5 days.

### Identification of *Legionella*isolates

*Legionellae* cannot grow on substrates lacking cysteine [23]. Therefore, samples were plated on media with (BCYE, bioMérieux) and without cysteine (Columbia Blood agar base, Oxoid): colonies not growing on media without cysteine were considered to be *Legionella* strains and were further identified by MALDI-TOF mass spectrometry [24] or by slide agglutination (SLIDEX® Legionella; bioMérieux). *Legionella pneumophila* strains were characterised by indirect immunofluorescence assay, using the monoclonal antibodies from the Dresden panel [25].

### Meteorological parameters

Local parameters such as air temperature [°C], relative humidity [%], dew point [hPa] and compost temperature [°C] at 30 cm depth in the oldest compost piles (Omniport 20, E + E Elektronik, Engerwitzdorf, Austria) were recorded during the sampling days. The dew point, which represents the temperature below which the water vapour in a volume of humid air at a constant pressure will condense into water, is a direct measure of the amount of water vapour in the air [26].

Wind speed was measured (Anemo Windmesser, Abisenvironment SA, Lonay, Switzerland) at a height of 1.8 m and wind direction was determined observing the dispersion of soap bubbles with the help of a compass.

### Meteorological data collection for the period 2007-2010

Meteorological data were obtained from two main weather stations in Ticino and two in the northern Cantons of Zurich and Aargau for the period between January 2007 and December 2010. The meteorological data evaluated were the monthly means of temperature of the air [°C], relative humidity of the air [%], pressure [hPa], wind speed [km/h] and total amount of precipitations [mm].

A one-way analysis of variance (ANOVA) was carried out with the means of the data of two weather stations of both regions to detect any statistically significant differences that could be due to differences in sampling years.

### Statistical analyses

The Simpson biodiversity index [SDI: 1-D′ = n*(n-1)/N*(N-1)] was computed for all compost samples investigated. A Chi-square test with Yates’ correction was carried out to determine whether or not there were significant differences among the compost samples from Ticino and from northern Swiss regions with regards to the presence of *Legionella* or FLA; and to compare proportion of contamination of composts with regard to *Legionella*-positive and FLA-positive samples. *P*-values <0.05 were considered statistically significant.

To assess differences among sampling sites and samples a canonical discriminant analysis was carried out using air temperature, relative humidity, dew point and wind direction, sampling hour and period (May-September), and the presence/absence of *Legionella pneumophila* 1 (Lp1), FLA supporting *Legionella* growth (Al), and both *L. pneumophila* (serogroups 1-15) and *L. bozemanii* (Lp-Lb) as independent variables. All statistical calculations were carried out using SPSS version 17.0 (SPSS Inc., Chicago, Illinois, USA).

Co-culture enrichment used in this study does not allow a quantitative assessment of the presence of *Legionella*, therefore all samples were classified only as positive or negative for the presence of *Legionella* and FLA.

## Results

### Geographic differences

#### Legionella spp

*Legionella* spp. were recovered from all composting facilities (CF) in Ticino and 3 out of 4 CF in northern Switzerland. In bioaerosols, *Legionella pneumophila* was present only in 50% of the facilities in southern Switzerland.

**Figure 1:**
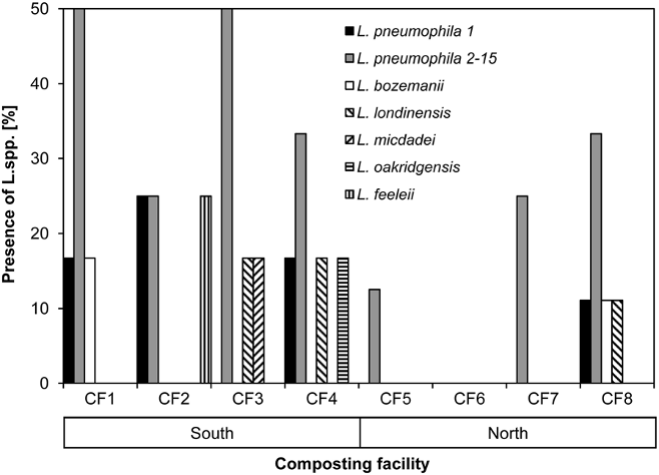
**Cumulative presence of** ***Legionella*** **spp. in compost and bioaerosol samples of composting facility (CF) from Swiss southern (CF1-4) and northern (CF5-8) alpine regions.**

*Legionella* were isolated from 13.3% of the bioaerosols and 100% of the compost samples in Ticino. By contrast, they were not present in any of the bioaerosols and were isolated only from 35.3% of the compost heaps in facilities located in the northern region (Figure 1). A statistically significantly higher proportion of *Legionella-*positive compost samples was found in CF in Ticino as compared to the north (*P* = 0.0146). For bioaerosol samples no differences were observed between the north and the south of Switzerland (*P* = 0.43).

All southern and 3 of 4 northern CF were colonised by *L. pneumophila. L. pneumophila* serogroup (sg) 1 was isolated in 3 of 4 facilities in Ticino but was present only in one in the northern Cantons. The Simpson biodiversity index of *Legionella* species was 0.75 in Ticino and 0.5 in the northern regions. Species diversity was higher in CF of Ticino, where 5 non-pneumophila species (*L. bozemanii*, *L. cincinnatiensis*, *L. feeleii*, *L. micdadei* and *L. oakridgensis*) were isolated as compared to two (*L. londiniensis* and *L. bozemanii*) identified in one centre in the north (Figure 1).

#### Free-living amoebae

FLA were isolated from 88.2% of the composts and 6.3% of the bioaerosols in the northern part of Switzerland, whereas in Ticino they were present in 64.1% of the composts and 13.9% of the bioaerosols studied. These differences, however, were not statistically significant (*P* = 0.89, *P* = 1).

**Figure 2:**
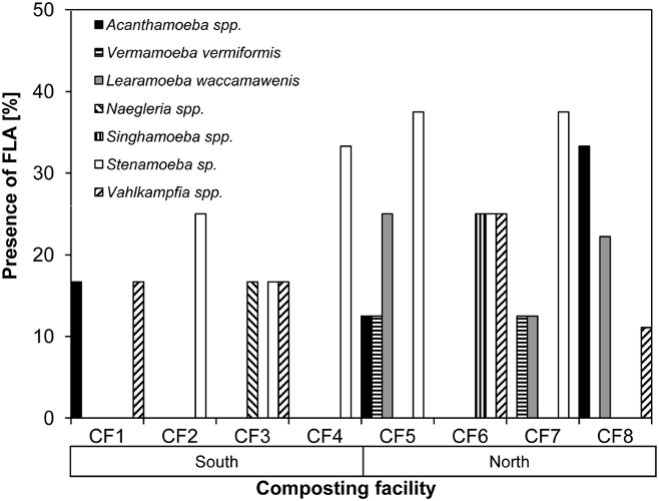
**Cumulative presence of FLA in compost and bioaerosol samples in composting facility (CF) from Swiss southern (CF1-4) and northern (CF5-8) alpine regions.**

Six genera of FLA (*Acanthamoeba*, *Vahlkampfia*, *Stenamoeba*, *Vermamoeba*, *Learamoeba* and *Singhamoeba*) were recovered from the compost in the northern regions (SDI: 0.80), as compared to 5 (*Acanthamoeba*, *Naegleria*, *Heterolobosea*, *Vahlkampfia* and *Stenamoeba*) in Ticino (SDI: 0.65) (Figure 2 and Additional file 1). Composts from long-term storage centres and CF in Ticino contained more FLA species compared to short-term storage centres (Table 2).

**Table 2:** **Presence of** ***Legionella*** **spp. and FLA in compost and bioaerosol samples among short-term (CS), long-term (CL) storage centres and composting facilities (CF) in southern alpine region**

	CS1	CS2	CS3	CL1	CL2	CF1	CF2	CF3	CF4
*L. pneumophila* 1	-	-	-	+	+	+	+	-	+
*L. pneumophila* 2-15	+	-	+	+	+	+	+	+	+
*L. bozemanii*	-	-	-	+	+	+	-	-	-
*L. londinensis*	-	-	-	-	-	-	-	+	+
*L. feeleii*	-	-	-	-	+	-	+	-	-
*L. oakridgensis*	-	-	-	-	+	-	-	-	+
*L. cincinnatiensis*	-	-	+	-	-	-	-	-	-
*L. micdadei*	-	-	-	-	-	-	-	+	-
*Stenamoeba* sp.	-	-	-	-	-	-	+	+	+
*Vahlkampfia* spp.	-	-	-	-	-	+	-	+	-
*Acanthamoeba* spp.	-	+	-	-	+	+	-	-	-
*Naegleria* spp.	+	-	-	+	-	-	-	+	-
*Heterolobosea* spp.	-	-	-	-	+	-	-	-	-

+: present; - absent.

### Differences among composting facilities, short-term and long-term storage centres

CF and long-term storage centres showed the highest *Legionella* spp. diversity (Table 2). The potentially pathogenic *L. pneumophila* sg 1 was present in all centres but CF3. *L. pneumophila* sg 1 was not isolated in short-term storage centres. *L. cincinnatiensis* was recovered only from one short-term storage centre. In Ticino all but one storage centre was positive for *L. pneumophila* sg 2-15.

### Differences in climatic conditions among sites

**Figure 3:**
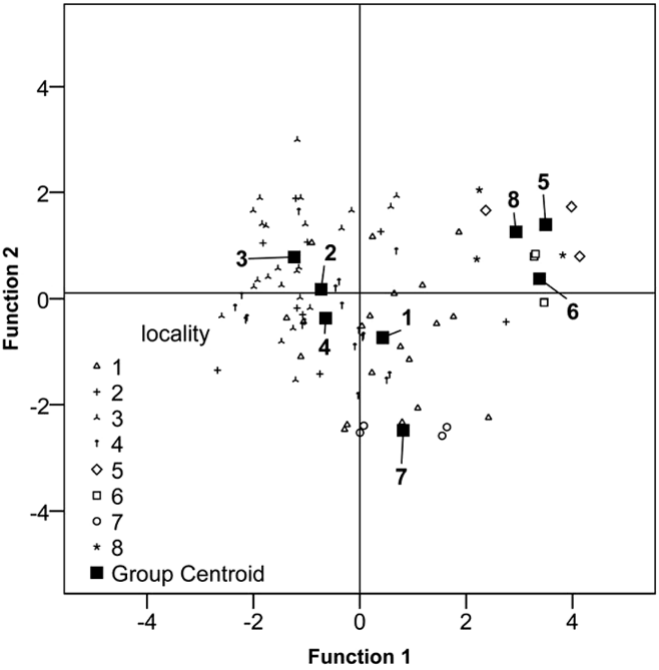
**Graphical plot of the results of discriminant analysis.** The centroids of the data originating from the southern facilities (1-4) are well grouped in the central part of the graph and separated from those of the northern facilities (5-8). Total variance explained by the model: 85.1%.

Discriminant analysis revealed that the centres investigated could be grouped according to their geographic origin. The first three canonical discriminant functions explain 85.1% of the variance of the data (Table 3). Centres CF5, CF6 and CF8 grouped together very closely, with CF7 in an isolated position, but separate from the southern alpine centres CF1-CF4 (Figure 3). The temperature of the air is responsible for a large load on the first function; relative humidity and dew point characterise the second and third functions (Table 3). The sampling days were not particularly windy and the very low wind speed could not be measured.

**Table 3:** **Percentage and number of samples (in parentheses) classified among the samples taken in the different composting facilities (CF1-CF8) by canonical discriminant analysis**

Locality*	Predicted group membership [%]							
Observed	CF1	CF2	CF3	CF4	CF5	CF6	CF7	CF8
CF1	60.0 (12)	5.0 (1)	5.0 (1)	15.0 (3)	0 (0)	5.0 (1)	10.0 (2)	0 (0)
CF2	11.1 (1)	33.3 (3)	11.1 (1)	44.5 (4)	0 (0)	0 (0)	0 (0)	0 (0)
CF3	0 (0)	4.2 (1)	87.5 (21)	8.3 (2)	0 (0)	0 (0)	0 (0)	0 (0)
CF4	27.8 (5)	0 (0)	11.1 (2)	61.1 (11)	0 (0)	0 (0)	0 (0)	0 (0)
CF5	0 (0)	0 (0)	0 (0)	0 (0)	100 (3)	0 (0)	0 (0)	0 (0)
CF6	0 (0)	0 (0)	0 (0)	0 (0)	0 (0)	100 (4)	0 (0)	0 (0)
CF7	0 (0)	0 (0)	0 (0)	0 (0)	0 (0)	0 (0)	100 (4)	0 (0)
CF8	0 (0)	0 (0)	0 (0)	0 (0)	0 (0)	0 (0)	0 (0)	100 (4)

72.1% are correctly classified (predicted) by the analysis.

*CF1-4 = Swiss southern composting facilities. CF5-8 = Swiss northern composting facilities.

### Meteorological characterisation of the period 2007-2010

We have also analysed the meteorological situation in the period 2007-2010. The monthly means of both regions (Additional file 2) show that climate is almost stable because no significant year-to-year differences occurred during the three sampling period. The *P*-value of one-way ANOVA of the monthly means of meteorological data from two main weather stations in Ticino and in northern Switzerland are not statistically significant: temperature (*P* = 0.97 in the North vs. *P* = 0.95 in Ticino), relative humidity (*P* = 0.95 vs. *P* = 0.17), wind speed (*P* = 0.90 vs. *P* = 023), precipitation (*P* = 0.19 vs. *P* = 0.83) and pressure (*P* = 0.11 vs. *P* = 0.11).

## Discussion

Our study shows that southern and northern compost heaps host different populations of *Legionella* and FLA, and the *Legionella* species richness is influenced by the climate and geography of the two regions. The duration of compost storage also influenced the degree of contamination of *Legionella* and FLA communities.

Potential human pathogenic strains of *L. bozemanii*, *L. cincinnatiensis*, *L. feeleii*, *L. micdadei*, *L. londiniensis* and *L. oakridgensis* were isolated mainly from samples collected in the southern part of Switzerland. The observed number of species (S), however, is not a reliable index of richness because it is influenced by the evenness: the more reliable Simpson biodiversity index was 25% higher in Ticino compared to the northern region. Ticino hosts a higher diversity of *Legionella* compared to the north, where only *L. bozemanii* and *L. londiniensis* were recovered. *L. longbeachae*, reported in other studies [10], was not detected in our samples. In Switzerland, *L. longbeachae* has so far been reported only once from potting soils [12] and from compost [21]. *L. pneumophila* sg 1 was isolated from compost in 75% of the southern Swiss facilities. These results support the hypothesis of compost being a potential source of *Legionella* causing LD [2].

Discriminant analysis show a trend towards separation of CF according to their geographic location, with 72.1% of the cases correctly classified (Table 3); this supports the hypothesis that climate and possibly geographic factors influence compost contamination by *Legionella*. North-south differences, as shown by the canonical discriminant analysis, were expected if considering the climate features of the two regions [15]. The most important discriminating factors are relative humidity, temperature and dew point. Our results are consistent with previously published observations [27, 28] that identified a relationship between occurrence of cases of LD and precipitation and increased humidity.

In the southern alpine region the normal relative humidity values vary between 62.8% and 68.9% from April to August, with a July monthly mean of 65.3%. In the northern alpine regions the values may exceed 72% [15]. A previous study showed that *L. pneumophila* is more viable in the aerosols at 65% than at 72% relative humidity [29], meaning that *Legionella* survival is more likely in the southern alpine region. The relative humidity is indirectly proportional to temperature and for this reason the values are higher in the northern alpine region. We have observed that vapour pressure is a better discriminating factor than humidity [30]. Vapour pressure is usually higher in Ticino from April to November, being e.g. 16 hPa in August, a month during which many LD cases are recorded [14, 15], as opposed to 14.5 hPa in the north. The climate characteristics in each region could thus play an important role in the bioaerosol stability and survival of *Legionella* and as a consequence in the transmission of LD.

More genera of FLA were recovered from the composts in the northern regions compared to Ticino. FLA may protect intracellular *Legionella* from adverse ecological factors, such as environmental conditions, nutrient starvation, biocides and predation [5]; FLA known to favour the intracellular growth of *Legionella* (i.e. *Acanthamoeba* spp., *Naegleria* spp., *Vahlkampfia* spp and *Vermamoeba vermiformis*) were isolated in both regions [3]. However, the presence of *Legionella* in compost in the south and in the north was statistically not correlated with the presence of FLA. These results must be taken with caution because of the rather small sample size and the limited number of isolated FLA.

FLA richness is apparently favoured by a combination of climatic factors [31], because more species of FLA were recovered in the northern composts, but the results may have been influenced by the low number of samples and replicates. The differences in *Legionella* diversity and FLA diversity, on the other hand, could be a random finding.

Long-term storage centres and CF did not show any differences in the variety of *Legionella* spp. and FLA spp. Short-term and long-term storage centres were very different with regards to contamination by *Legionella* spp. Duration of outdoor storage in centres, with or without structured management of green waste, is most likely influencing colonisation of the heaps by *Legionella* (Table 2). The differences seen between southern and northern CF and among storage centres may have different explanations. Green material in CF was described by Casati et al. [11] to be *Legionella*-free.

The green material composted, however, could in fact be contaminated, the concentration of *Legionella* cells, however, being below the limit of detection, which is 10^2^ CFU per g of compost [20].

The differences in the climate between southern and northern regions could also influence the vegetation and thus the composition of the material collected in the compost centres. However, as the collected green waste mainly comes from gardens, this difference may have limited influence on the final compost composition.

Previously published data suggest that, once contaminated, compost heaps may become an optimal reservoir [11]. The precise mode of contamination remains controversial, but rain [11], bioaerosol and dust moved by wind may be causal factors. Even though the presence of *Legionella* in rainwater was not assessed in this study, viable *Legionella* were recently recovered in samples from pluvial floods, puddles on asphalt roads and roof-harvested rainwater tanks. It would be interesting to assess if there is an increase in *Legionella* concentrations in samples collected near composting heaps compared to rainwater collected elsewhere [32–35].

Bioaerosols may be released from the compost passively by evaporation and heat, or actively through human activities in the facilities [19]. Compost is generally reused in agriculture, gardens and flowerbeds, or mixed with soil in potting soil. Recycling of the compost could introduce potential pathogenic *Legionella* into the soil or potting soil; handling of these products could generate bioaerosols or dust [36] that may be potential sources of *Legionella* infections [9, 37].

This study suffers from three limitations. One is the small number of samples and CF analysed. We used stringent criteria to choose the facilities, because we aimed at investigating open field facilities with complete and structured management of the green waste. In the southern alpine region structured green management is not common; therefore, we decided to consider also long- and short-term green waste storage centres. In the northern alpine region, open field facilities are increasingly replaced by indoor composting to exploit biogas production. We did not analyse the situation in these centres because they should not pose human health threat for community-acquired LD. This limited the availability of additional facilities for our investigations. Further, *Legionella* in compost was analysed by culture and co-culture (amoebal enrichment method); in contrast, bioaerosols were analysed only by co-culture.

The results of the analysis of bioaerosols by traditional culture would have been an interesting addition to the study. In fact, co-culture performed with only one amoebal strain may have selected for particular species and may have given only a partial picture of the bacterial population within the composts.

In a pilot study, 20 bioaerosol samples were analysed by culture and co-culture in parallel (Additional file 3). Nine samples were positive only by co-culture analysis and this prompted us to analyse bioaerosol samples only by co-culture, because we assumed that the amount of *Legionella* cells present in the bioaerosol was too low.

Finally logistic problems hindered us to sample the different facilities during the same year and we had to carry out our sampling in different years, thus introducing a confounding factor (time) in our analysis. The one-way ANOVA carried out on the meteorological data, on the other hand, did not reveal statistically significant differences among the years with regards to the parameters investigated. Analysis of data from multiple years are thus unlikely to have influenced our observations. Sampling was also performed during the same period of the year, in summer, with constant conditions between the three years in both regions. We are thus confident that different sampling times have not influenced relevantly the outcome of the study.

## Conclusions

In conclusion, CF of southern and northern Swiss regions host different populations of *Legionella* and FLA. Composts and bioaerosols in Ticino are frequently contaminated by *Legionella* and compost contamination by *Legionella* and FLA seems to increase with increasing storage time. These differences are most likely influenced by the different climates in the two regions. Future studies should address the role that CF may have in the spread of *Legionella* to better understand the importance of composting facilities in the transmission of LD.

## Electronic supplementary material

Additional file 1: **Results of BLAST analysis for 18S rRNA gene sequences of recovered from FLA strains.** BLAST analysis was used to determine the level of 18S rRNA gene sequence homology with the most similar GenBank sequence. ^*a*^18S rRNA gene query coverage/homology with closest GenBank described species. (DOCX 13 KB)

Additional file 2: **Mean meteorological data from two main weather stations each in Ticino and in the Cantons of Zurich and Aargau for the period between January 2007 and December 2010.** (DOCX 23 KB)

Additional file 3: **List of all** ***Legionella*** **spp. recovered from bioaerosol samples analysed in parallel by culture and co-culture.** CS; short-term green wastes storage centre, CL; long-term green wastes storage centre, CF; composting facility, Lp1; *L. pneumophila* serogroup 1, Lp2-15; *L. pneumophila* serogroups 2-15, -; negative sample. (DOCX 18 KB)
