# Supplementary material for: Influence of climate and geography on the occurrence of Legionella and amoebae in composting facilities
Source: BMC Res Notes. 2014 Nov 24;7:831. doi: 10.1186/1756-0500-7-831 (PMC4289342; doi:10.1186/1756-0500-7-831)
Supplement: Supplementary file 1 — Additional file 1: Results of BLAST analysis for 18S rRNA gene sequences of recovered from FLA strains. BLAST analysis was used to determine the level of 18S rRNA gene sequence homology with the most similar GenBank sequence. a18S rRNA gene query coverage/homology with closest GenBank described species. (DOCX 13 KB) [file 13104_2013_3430_MOESM1_ESM.docx]

| **Taxon (GenBank Accession No.)** | **Strain (GenBank Accession No.)** | **Origin** | **Query coverage/ Nucleotide identity*^a^*** |
| --- | --- | --- | --- |
| *Acanthamoeba polyphaga* (U07415) | CF1-144 (KC346958) | compost | 100%/99% |
| *Acanthamoeba polyphaga* (U07415) | CL2-C58 (KC346959) | compost | 98%/99% |
| *Acanthamoeba hatchetti* (JF508857) | CS2-01 (KC346960) | aerosol | 100%/100% |
| *Heterolobosea* sp. (DQ388519) | CL2-34 (KC346961) | aerosol | 100%/97% |
| *Naegleria* sp. (KC164220) | CS1-C06 (KC346962) | compost | 99%/99% |
| *Naegleria* sp. (HQ007042) | CL1-C05 (KC346963) | compost | 100%/100% |
| *Vahlkampfia avara* (AJ224886) | CF1-144 (KC164241) | compost | 100%/99% |
| *Vahlkampfia avara* (AJ224886) | CF3-154 (KC164242) | compost | 100%99% |
| *Stenamoeba* sp. (EU377587) | CF5-304 (KC346964) | compost | 71%/100% |
| *Stenamoeba limacina* (GU810183) | CF6-313 (KC346965) | compost | 72%/99% |
| *Stenamoeba* sp. (EU377587) | CF7-321 (KC346969) | compost | 80%/100% |
| *Vermamoeba vermiformis* (FJ628003) | CF5-306 (KC346966) | compost | 98%/100% |
| *Singhamoeba horticola* (KC164229) | CF6-312 (KC346967) | compost | 97%/99% |
| *Learamoeba waccamawensis* (KC164239) | CF8-329 (KC346968) | compost | 99%/99% |
