# Supplementary material for: Influence of climate and geography on the occurrence of Legionella and amoebae in composting facilities
Source: BMC Res Notes. 2014 Nov 24;7:831. doi: 10.1186/1756-0500-7-831 (PMC4289342; doi:10.1186/1756-0500-7-831)
Supplement: Supplementary file 2 — Additional file 2: Mean meteorological data from two main weather stations each in Ticino and in the Cantons of Zurich and Aargau for the period between January 2007 and December 2010. (DOCX 23 KB) [file 13104_2013_3430_MOESM2_ESM.docx]

|  |  | **North** |  |  |  |  | **South** |  |  |  |  |
| --- | --- | --- | --- | --- | --- | --- | --- | --- | --- | --- | --- |
|  | **Month** | **2007** | **2008** | **2009** | **2010** | **P-Value** | **2007** | **2008** | **2009** | **2010** | **P-Value** |
| **Temperature** | 1 | 4.85 | 2.65 | -1.75 | -1.45 | 0.97 | 6.35 | 4.65 | 2.6 | 1.8 | 0.95 |
|  | 2 | 4.8 | 3.4 | 0.55 | 0.85 |  | 7.2 | 5.85 | 4.85 | 4 |  |
|  | 3 | 5.75 | 5 | 4.75 | 4.7 |  | 10.05 | 9.2 | 9.15 | 7.5 |  |
|  | 4 | 13.6 | 8.4 | 11.9 | 10.3 |  | 16.15 | 11.3 | 12.65 | 12.2 |  |
|  | 5 | 15.2 | 15.55 | 15.65 | 12.15 |  | 17.5 | 16 | 18.7 | 14.9 |  |
|  | 6 | 17.95 | 17.65 | 17.1 | 17.3 |  | 19.7 | 19.85 | 20.4 | 20.05 |  |
|  | 7 | 18.1 | 18.75 | 18.6 | 20.25 |  | 22.35 | 21.3 | 22.05 | 24.35 |  |
|  | 8 | 17.6 | 17.9 | 19.5 | 17.45 |  | 20.35 | 21.6 | 22.85 | 20.9 |  |
|  | 9 | 13.25 | 12.65 | 15.35 | 13.2 |  | 16.95 | 16.3 | 18.65 | 17.05 |  |
|  | 10 | 9.4 | 10.05 | 9.35 | 8.8 |  | 13.45 | 13.3 | 13.1 | 11.6 |  |
|  | 11 | 3.05 | 4.25 | 6.75 | 5.4 |  | 7.75 | 7.65 | 8.55 | 7.4 |  |
|  | 12 | 1.05 | 0.8 | 1.15 | -0.8 |  | 4.45 | 3.9 | 3.3 | 2.3 |  |
| **RH** | 1 | 79.3 | 83.9 | 85.65 | 85.15 | 0.95 | 69.8 | 74.6 | 75.95 | 70.9 | 0.17 |
|  | 2 | 80.55 | 77.15 | 81.25 | 78.45 |  | 68.55 | 67.35 | 58.5 | 68.4 |  |
|  | 3 | 76.25 | 72.95 | 75.65 | 70.15 |  | 55.3 | 50.9 | 53.8 | 63.95 |  |
|  | 4 | 61.8 | 77 | 67.25 | 64.9 |  | 60.85 | 62.9 | 69.1 | 61.95 |  |
|  | 5 | 70.95 | 68 | 72.25 | 77.3 |  | 61.8 | 73.4 | 58.65 | 68.15 |  |
|  | 6 | 74.65 | 75.8 | 71.9 | 73.7 |  | 73.6 | 74.45 | 61.65 | 67.2 |  |
|  | 7 | 74.4 | 71.45 | 76.75 | 73.4 |  | 54.7 | 66.9 | 63.85 | 58.4 |  |
|  | 8 | 79.4 | 78.2 | 76.65 | 79.1 |  | 70 | 67.25 | 68.3 | 68.1 |  |
|  | 9 | 81.3 | 82.6 | 80.75 | 79.7 |  | 63.6 | 74.4 | 73.5 | 69.35 |  |
|  | 10 | 83.6 | 85.7 | 83.15 | 83.5 |  | 70.25 | 76.9 | 66.55 | 75.55 |  |
|  | 11 | 83.6 | 88.2 | 87.8 | 84.5 |  | 58.35 | 77.5 | 80.3 | 79.85 |  |
|  | 12 | 85.5 | 87.4 | 84.55 | 86.05 |  | 61.4 | 77.4 | 67.9 | 66.5 |  |
| **Wind speed** | 1 | 68.75 | 53.3 | 35.65 | 30.35 | 0.90 | 67.3 | 148.75 | 39.25 | 40.95 | 0.23 |
|  | 2 | 69.8 | 32.95 | 53.25 | 45.45 |  | 24.75 | 38.05 | 138.2 | 84.75 |  |
|  | 3 | 94.4 | 89.2 | 127.65 | 51.1 |  | 58.65 | 83.45 | 125.6 | 131.55 |  |
|  | 4 | 11.95 | 178.25 | 19.95 | 27.3 |  | 19.45 | 233.25 | 284.75 | 84.85 |  |
|  | 5 | 145.55 | 44.9 | 90 | 138.9 |  | 195.4 | 277.35 | 58 | 375.75 |  |
|  | 6 | 115.9 | 66.15 | 112.6 | 117.3 |  | 261.95 | 159.3 | 206.1 | 174.25 |  |
|  | 7 | 169.35 | 114.8 | 169.25 | 133.25 |  | 93.45 | 321.95 | 373.2 | 114.4 |  |
|  | 8 | 210.3 | 147.15 | 60.35 | 141.85 |  | 314.2 | 101.65 | 138.6 | 206.45 |  |
|  | 9 | 57.8 | 102.1 | 53.4 | 55.95 |  | 161 | 270.3 | 120.95 | 152.55 |  |
|  | 10 | 15.75 | 134.65 | 42.1 | 43.9 |  | 13.3 | 209.8 | 105.05 | 197 |  |
|  | 11 | 57.05 | 37.55 | 116.3 | 77.4 |  | 103.1 | 257.85 | 174.35 | 246.05 |  |
|  | 12 | 98.85 | 77.05 | 108.55 | 92.6 |  | 10.95 | 166.15 | 128.8 | 128.45 |  |
| **Precipitation** | 1 | 8.6 | 5.4 | 5.05 | 4.85 | 0.19 | 4.1 | 3.6 | 3.05 | 4.3 | 0.83 |
|  | 2 | 6.3 | 5.4 | 5.05 | 5.95 |  | 3.95 | 4.15 | 6.3 | 4.35 |  |
|  | 3 | 5.75 | 9.7 | 7.2 | 7.2 |  | 6.5 | 7.55 | 6.65 | 5.75 |  |
|  | 4 | 4.35 | 6.3 | 4.85 | 5.4 |  | 5.95 | 6.45 | 4.85 | 5.95 |  |
|  | 5 | 7.05 | 5.05 | 4.65 | 5.4 |  | 5.95 | 4.85 | 5.55 | 6.3 |  |
|  | 6 | 5.55 | 4.65 | 5.4 | 4.5 |  | 5.2 | 4.5 | 6.5 | 5.75 |  |
|  | 7 | 5.95 | 4.65 | 4.85 | 3.75 |  | 5.75 | 5.6 | 5.2 | 6.15 |  |
|  | 8 | 4.15 | 4.3 | 3.8 | 4.65 |  | 5 | 4.85 | 4.7 | 5.4 |  |
|  | 9 | 4.3 | 4.35 | 3.6 | 3.4 |  | 5.9 | 5.05 | 4.35 | 5 |  |
|  | 10 | 4.5 | 4.5 | 3.8 | 4 |  | 4.85 | 4.35 | 5.25 | 4.15 |  |
|  | 11 | 6.5 | 4.65 | 5.2 | 5.4 |  | 5.6 | 4.15 | 3.4 | 3.95 |  |
|  | 12 | 7.75 | 5.4 | 5.4 | 4.65 |  | 4.3 | 3.95 | 4.3 | 5.25 |  |
| **Pressure** | 1 | 973.4 | 972 | 965.7 | 964.55 | 0.11 | 980.85 | 982.45 | 973.75 | 972.45 | 0.11 |
|  | 2 | 965.1 | 976.8 | 965.4 | 957.2 |  | 974.25 | 986.7 | 972.25 | 966.55 |  |
|  | 3 | 967.4 | 960.9 | 966.6 | 968.15 |  | 974.35 | 967.55 | 972.35 | 976.65 |  |
|  | 4 | 969.5 | 962.5 | 964.35 | 969.1 |  | 977.85 | 970.95 | 974.6 | 977.4 |  |
|  | 5 | 963.95 | 964.95 | 970.3 | 965.15 |  | 971.95 | 975.25 | 978.45 | 971.95 |  |
|  | 6 | 965.35 | 967.95 | 967.65 | 966.4 |  | 973.75 | 975.6 | 974 | 973.45 |  |
|  | 7 | 967.65 | 968.1 | 968.6 | 969.5 |  | 974.85 | 975.25 | 976.3 | 976.35 |  |
|  | 8 | 967.15 | 967.55 | 969.9 | 967.95 |  | 974.75 | 974.95 | 977.65 | 975 |  |
|  | 9 | 971.25 | 969.1 | 971.25 | 967.95 |  | 977.85 | 976.6 | 979.05 | 975 |  |
|  | 10 | 973.3 | 969.85 | 969.6 | 966.1 |  | 980.4 | 978.65 | 976.85 | 975.4 |  |
|  | 11 | 970.85 | 966.55 | 963.95 | 959.5 |  | 977.5 | 975.9 | 974.6 | 968.8 |  |
|  | 12 | 974.55 | 968.85 | 960.3 | 963.35 |  | 982.45 | 977.2 | 969.2 | 971.85 |  |
