# Supplementary material for: Influence of climate and geography on the occurrence of Legionella and amoebae in composting facilities
Source: BMC Res Notes. 2014 Nov 24;7:831. doi: 10.1186/1756-0500-7-831 (PMC4289342; doi:10.1186/1756-0500-7-831)
Supplement: Supplementary file 3 — Additional file 3: List of all Legionella spp. recovered from bioaerosol samples analysed in parallel by culture and co-culture. CS; short-term green wastes storage centre, CL; long-term green wastes storage centre, CF; composting facility, Lp1; L. pneumophila serogroup 1, Lp2-15; L. pneumophila serogroups 2-15, -; negative sample. (DOCX 18 KB) [file 13104_2013_3430_MOESM3_ESM.docx]

| **Sample** | **Centre** | **Culture** | **Co-culture** |
| --- | --- | --- | --- |
| **Ae3** | CS1 | - | Lp2-15 |
| Ae1 | CS2 | - | - |
| Ae2 | CS2 | - | - |
| **Ae4** | CS3 | - | Lp1, Lp2-15 |
| **Ae5** | CL1 | - | Lp1, Lp2-15 |
| Ae8 | CL1 | - | - |
| Ae12 | CF1 | - | - |
| Ae13 | CF1 | - | - |
| **Ae26** | CF3 | - | Lp2-15, *L. bozemanii* |
| **Ae27** | CF3 | - | Lp2-15 |
| **Ae28** | CF3 | - | Lp2-15 |
| **Ae29** | CF3 | - | Lp2-15 |
| **Ae6** | CF4 | - | Lp2-15 |
| **Ae7** | CF4 | - | Lp2-15 |
| Ae9 | CF4 | - | - |
| Ae10 | CF4 | - | - |
| Ae11 | CF4 | - | - |
| Ae31 | CF4 | - | - |
| Ae32 | CF4 | - | - |
| Ae33 | CF4 | - | - |

CS; short-term green wastes storage centre, CL; long-term green wastes storage centre, CF; composting facility, Lp1; *L. pneumophila* serogroup 1, Lp2-15; *L. pneumophila* serogroups 2-15, -; negative sample.
